# Protective Effects of Necrostatin-1 against Concanavalin A-Induced Acute Hepatic Injury in Mice

**DOI:** 10.1155/2013/706156

**Published:** 2013-10-01

**Authors:** Yingqun Zhou, Weiqi Dai, Chunlei Lin, Fan Wang, Lei He, Miao Shen, Ping Chen, Chenfen Wang, Jie Lu, Ling Xu, Xuanfu Xu, Chuanyong Guo

**Affiliations:** Department of Gastroenterology, The Tenth People's Hospital of Tongji University, Shanghai 200072, China

## Abstract

*Objective.* Necrostatin-1 (Nec-1) inhibits receptor-interacting protein 1 (RIP1) kinase and programmed necrosis. This study was designed to examine the protective effects and mechanisms of Nec-1 in concanavalin A- (ConA-) induced hepatitis in mice. *Methods.* C57BL/6 mice were exposed to ConA via tail vein injection and injected intraperitoneally with Nec-1 or vehicle. Levels of serum liver enzymes and histopathology were determined. Levels of inflammatory cytokines with ConA-induced hepatitis were determined with real-time polymerase chain reaction (real-time PCR). The expression of TNF-**α**, RIP1, and LC3 was detected with immunohistochemical staining. The expression of TNF-**α**, IFN-**γ**, IL2, IL6, caspase 3, RIP1, beclin-1, and LC3 protein was assessed by immunofluorescence and western blotting. Autophagosomes were observed with transmission electron microscopy (TEM). *Results.* Amelioration in liver functions and histopathological changes and the suppression of inflammatory cytokine production were observed in Nec-1-injected mice. Western blotting analysis showed that the expression of TNF-**α**, IFN-**γ**, IL2, IL6, and RIP1 was significantly reduced in the Nec-1-injected mice, which was confirmed by immunofluorescence and immunohistochemistry. Autophagosome formation was significantly reduced by Nec-1 treatment, as the expression of beclin-1 and LC3, determined with immunofluorescence and western blotting. *Conclusion.* These results demonstrate that Nec-1 prevents ConA-induced liver injury via RIP1-related and autophagy-related pathways.

## 1. Introduction

 Liver diseases caused by various factors, including viruses, chemicals, drugs, alcohol, genetic factors, or a patient's own immune system, represent a significant public health problem because liver damage can progress to liver failure [1]. Liver inflammation may be acute, flaring up and resolving within a few weeks to months, or chronic, enduring over many years. Chronic hepatitis can simmer for 20 years or more before causing significant symptoms related to advanced liver damage, including cirrhosis (scarring and liver failure), liver cancer, or death [2]. Hepatitis represents a worldwide health problem in humans, for which the currently available pharmacological treatments are inadequate. However, the development of new drugs requires proper animal models relevant to human hepatitis [2, 3]. Most liver diseases, such as viral hepatitis, autoimmune hepatitis, primary biliary cirrhosis, primary sclerosing cholangitis, and liver allograft rejection, are caused by activated T lymphocytes, which infiltrate and destroy the liver parenchyma, leading to liver injury [4]. The injection of mice with the T-cell mitogenic plant lectin concanavalin A (ConA) results in the polyclonal activation of T lymphocytes, inducing a liver-specific inflammatory response that mimics activated T-cell-mediated hepatitis [5]. ConA-induced hepatitis is an ideal animal model that simulates the pathophysiology of human viral and autoimmune hepatitis and has been used extensively to clarify various aspects of human T-cell-mediated liver diseases [5–11].

 Two mechanisms, apoptosis and necrosis, are frequently involved in acute and chronic liver injury. Apoptosis is a precisely regulated and genetically determined process that can be induced by death-receptor-mediated extrinsic pathways or by intrinsic mechanisms activated by intracellular stress [12]. Apoptosis is executed by a group of intracellular cysteine proteases called “caspases” [13]. With caspase activation and the subsequent cleavage of their intracellular substrates, cells break into small membrane-wrapped vesicles known as “apoptotic bodies” [14]. In contrast, necrosis is associated with organelle swelling, cytoplasmic membrane breakdown, and ensuing inflammatory responses [15].

 Recently, a novel type of cell death called “necroptosis” was identified by Degterev et al. [16]. These researchers used tumor necrosis factor-*α* (TNF-*α*) and Fas ligand to induce necroptosis in different cells, including Jurkat cells and mouse embryonic fibroblasts [16, 17]. Necroptosis, or programmed necrosis, is a caspase-independent mode of cell death that prevails when caspases are not activated or are inhibited [18]. The term “necroptosis” refers only to regulated necrotic cell death, which is mediated by receptor-interacting protein 1 (RIP1) kinase activity. Therefore, by definition, necroptosis is inhibited by necrostatin-1 (Nec-1), 5-(1H-indol-3-ylmethyl)-3-methyl-2-sulfanylideneimidazolidin-4-one, a small-molecule inhibitor of programmed cell necrosis that has shown promise as a neuroprotectant in adult rodent models of myocardial ischemia and traumatic and ischemic brain injury [16, 19]. Nec-1 specifically inhibits necroptosis but not apoptosis. The inhibition of necroptosis by Nec-1 reduced the infarct volume and ameliorated the neurological deficit in a mouse model of middle cerebral artery occlusion [16]. Little is known about the protein involved in and regulating cell signaling during necroptosis. However, recent studies have revealed that the receptor-interacting serine-threonine kinase family member RIP plays a crucial role in regulating the switch between apoptosis and necroptosis [20]. When caspases are inhibited, stimulation with TNF-*α* triggers necroptosis, mediated by RIP1 and RIP3 [21]. The mechanism by which RIP kinases orchestrate apoptotic or necroptotic cell death has been studied intensively [22, 23].

Autophagic cell death is another important physiological cell death process. This type of cell death is characterized by the massive degradation of the cellular contents, including essential organelles such as the mitochondria, in membrane vesicles called “autophagosomes,” which then fuse with lysosomes to become autolysosomes [24, 25]. Autophagy allows a cell to survive in the harsh environment with self-help behavior. To a certain extent, autophagic cells play a protective role but, beyond this point, result in tissue damage. There is increasing evidence that blocking the cell death pathways can significantly reduce the organ and tissue damage caused by inflammation [26, 27]. Therefore, reducing liver cell death is considered to be a reasonable treatment for potential acute liver injury. Nec-1 protects cells by inhibiting RIP1 kinase activity and possible downstream anti-inflammatory effects and perhaps by other mechanisms [28]. Currently, the mechanism by which Nec-l inhibits ConA-induced acute liver injury and cell death is unclear. With the establishment of animal models of experimental liver injury, it is possible to explore the mechanism of Nec-1's action, to screen drugs, to treat acute liver injury, to examine the pathophysiological changes that occur in acute liver injury, and to significantly improve the level of clinical treatment available.

Therefore, this study is designed to assess the role of RIP1 and autophagy in the protective effect of ConA-induced liver injury. We show that preconditioning of Nec-1 can inhibit the RIP1 kinase pathways and reduce the ConA-induced injury in vivo. In addition, we also found that Nec-1 treatment can degrade the level of autophagy in hepatocytes after ConA-induced injury. Furthermore, we investigated the possible mechanisms underlying this protective effect.

## 2. Materials and Methods

### 2.1. Reagents

 ConA, dimethyl sulfoxide (DMSO), and RIP1 were purchased from Sigma Aldrich (St. Louis, MO, USA). Antisera against microtubule-associated protein light chain 3 (LC3) and beclin-1 were purchased from Abcam (Abcam Cambridge, MA, USA). Nec-1 was obtained from Chembridge Corporation (San Diego, CA, USA). Anti-RIP1 (0.2 *μ*g/mL; H-207, Santa Cruz Biotechnology, Santa Cruz, CA, USA) is a rabbit polyclonal antibody that does not cross react with RIP2 or RIP3. Anti-TNF-*α* (0.1 *μ*g/mL; Millipore; Billerica, MA, USA) is a rabbit polyclonal antibody raised against recombinant mouse TNF-*α*. Other antibodies used in the present study include goat anti-IL2,6 and goat anti-caspase polyclonal antibodies (Santa Cruz Biotechnology). The RNA PCR kit was purchased from Takara (Takara Biotechnology, Dalian, China). Dulbecco's modified Eagle's medium and fetal bovine serum were obtained from Invitrogen Corporation (Carlsbad, CA, USA).

### 2.2. Animals

 Male C57BL/6 mice (6–8 weeks old, 20 ± 2 g) were obtained from Shanghai SLAC Laboratory Animal Co. Ltd (Shanghai, China). They were housed in plastic cages with controlled light-dark cycles and fed a standard diet with water, in a controlled-temperature (25 ± 1°C) and humidity (50 ± 5%) environment. The experiments were performed according to the National Institutes of Health Guidelines for the Care and Use of Laboratory Animals and were approved by the Committee on the Ethics of Animal Experimentation of Hirosaki University.

### 2.3. Ethics Statement

The mice were housed and maintained under specific-pathogen-free conditions in the Animal Resource Facility of University of Tongji, and all experiments were preapproved by the Institutional Animal Care and Use Committee of that institution (IACUC approval no. SYXK (Shanghai) 2011-0111).

### 2.4. Drug Administration

 ConA was dissolved in pyrogen-free normal saline solution (NSS) at a concentration of 2.5 mg/mL and injected intravenously at a dose of 20 mg/kg body weight to induce hepatitis, as previously described [5]. Nec-1 was dissolved in 0.5% DMSO at a concentration of 5 mg/mL and was injected intraperitoneally at a dose of 1.8 mg/kg body weight. The mice were randomly divided into four groups: the normal control group was injected via the tail vein with NSS alone; the vehicle group was injected intraperitoneally with DMSO without ConA; the model ConA group was injected via the tail vein with ConA without Nec-1 treatment; the Nec-1 pretreatment group was injected intraperitoneally with Nec-1 1 h before the ConA challenge. At the end of the experiments, the mice were killed at the indicated time points after ConA injection, and their serum and liver tissue samples were collected. At the same time, we calculated the differences in the mortality of the mice.

### 2.5. Biochemical Analysis: Analysis of Liver Transaminase Levels

 Hepatocyte damage was assessed at the indicated time points after ConA injection with the measurement of plasma alanine transaminase (ALT) and aspartate transaminase (AST) activities using an automated clinical analyzer (Olympus AU1000; Olympus, Tokyo, Japan). The blank group was injected with saline via the tail vein. The other groups of animals were subjected to a tail vein injection of ConA (20 mg/kg), and their orbital blood was collected after 8 h, 12 h, and 20 h. The animals were killed after anesthetization with an intraperitoneal injection of sodium pentobarbital (62.5 mg/kg).

### 2.6. Histopathology and Immunohistochemistry

 The mouse livers were fixed in formalin and embedded in paraffin. Paraffin sections (5 *μ*m thick) were stained with hematoxylin-eosin (H&E) and examined by light microscopy for inflammation and tissue damage by one blinded pathologist. The percentage injury was determined by measuring the total area of portal inflammation, lobular inflammation, and necrosis and comparing it with the uninjured area in the corresponding region.

 The liver tissue specimens were cut into 4 *μ*m sections and dewaxed, hydrated, and pretreated with a heat-induced antigen retrieval technique. The sections were blocked and incubated overnight at 4°C with antibodies directed against RIP1, TNF-*α*, and LC3 at 1 : 100 dilutions and with a secondary antibody diluted 1 : 50 for 60 min at room temperature. Each antibody was diluted in Tris-buffered saline (TBS), 2% bovine serum albumin (BSA). The negative control antibodies consisted of species-matched immunoglobulin (Ig) fractions, which were also immunoglobulin G- (IgG-) subclass-matched where appropriate, used at the same dilution as the secondary antibodies. Color was developed with 3,3′-diaminobenzidine tetrachloride during incubation for 5–10 min. The specific staining with this substrate was observed under a light microscope [29].

 Staining was documented with a digital camera (Olympus) mounted on a microscope (Leica, Wetzlar, Germany) at ×200 magnification. Hepatocytes with brown staining in the cytoplasm demonstrated the normal localization of RIP1, TNF-*α*, and LC3. Ten separate high-power fields were chosen randomly in each section, and five mice from each group were examined.

### 2.7. Measurement of Serum Cytokines by Polymerase Chain Reaction (PCR)

 Total RNA was isolated and transcribed into cDNA using the RNeasy Mini Kit (Qiagen, Hilden, Germany) and the High Capacity cDNA Reverse Transcription Kit (Applied Biosystems, Foster City, CA, USA), respectively. The resulting cDNA was used as the template for PCR with primers specific for TNF-*α*, IFN-*γ*, IL2, IL4, IL6, IL10, and glyceraldehyde-3-phosphate dehydrogenase (GAPDH) (see Table 1). GAPDH was used as the internal control in the experiment.

### 2.8. Western Blotting Analysis

 Frozen livers were sonicated in phosphate-buffered saline (PBS) containing 0.1% Tween 20 and protease inhibitors. After centrifugation the supernatants were aspirated and their protein concentrations determined. An aliquot (90 *μ*g) of each liver lysate was separated on a 10% sodium dodecylsulfate-polyacrylamide gel and transferred onto a nitrocellulose membrane (Hybond ECL; Amersham Biosciences, Freiburg, Germany). The membranes were incubated in blocking buffer (5% nonfat milk powder in TBST [TBS with 0.1% Tween 20]) for 3 h and then incubated overnight at 4°C with gentle shaking with specific primary antibody directed against RIP1 (diluted 1 : 1000) and monoclonal anti-TNF-*α* (1 : 500), anti-IFN-*γ* (1 : 500), anti-IL2 (1 : 500), anti-IL6 (1 : 500), anti-LC3 (1 : 500), or anti-caspase-3 (1 : 1000) antibody. After the membrane was washed, it was incubated at room temperature for 1 h with a horseradish-peroxidase-labeled secondary antibody (Sigma-Aldrich). The membranes were visualized with enhanced chemiluminescence (SuperSignal; Pierce, Rockford, IL, USA).

### 2.9. Immunofluorescence

Fresh liver tissues collected from the mice were fixed in 4% paraformaldehyde on ice for 1 h. The fixed liver tissues were washed three times with PBS for 5 min on ice before they were dehydrated overnight in 30% sucrose (dissolved in PBS) at 4°C. The tissues were infiltrated with OCT for 2 h on day 2 and then frozen and stored at –80°C. Sections (5 *μ*m) were cut with a freezing microtome and stored at –20°C. Before analysis, the prepared sections were dried at room temperature for 5 min, and the OCT was dissolved in PBS for 5 min. The cell membranes were ruptured with 0.2% Triton X-100 at room temperature for 20 min. Nonspecific antigen-binding sites were blocked with 5% BSA, and the sections were then incubated overnight with antibody directed against TNF-*α*, RIP1, beclin-1, LC3, IL2, or IL6 (1 : 1000) at 4°C. After the samples were incubated with anti-rabbit antibody for 30 min on day 2, the cell nuclei were stained with DAPI (4,6-united amidine-2-phenylindole) (1 : 1000). All sections were observed with fluorescence microscopy.

### 2.10. Transmission Electron Microscopy

Mice were treated as described above, and the liver was flushed with 1 mL NSS and then perfused with 2 mL 4% glutaraldehyde in PBS and postfixed in 1% OsO_4_. Livers were sectioned and photographed using a transmission electron microscope (JEOL, JEM 1230) at 80 or 60 kV onto electron microscope film (Kodak, ESTAR thick base) and printed onto photographic paper.

### 2.11. Statistical Analysis

 The data are expressed as means ± standard deviations (SD). Differences among the experimental groups were determined with the unpaired Student's *t*-test or analysis of variance (ANOVA) followed by the Bonferroni post hoc test or Tukey's test when *F* was significant. All statistical analyses were performed with SPSS v13.0 statistical analysis software. Significance was established at a *P* value of less than 0.05.

## 3. Results

### 3.1. Survival Rates Were Improved by Injection of Nec-1

The blank control group mice had good statement; their activity, behavior, and vital signs were normal and no animal died. Two hours after injection with ConA, the model group showed reduced activity, apathy, vertical hair, and coiled body signs. At 12 h, mouse mortality was 13% in the model group, which differed significantly from that in the blank control group (*P* < 0.05; see Table 2).

Nec-1 reduced the mortality attributable to ConA-induced acute liver injury. The vehicle (DMSO) group and model (ConA) group showed similar symptoms. The intervention (Nec-1) group mice also displayed the symptoms and signs of acute liver injury, but they were alleviated relative to those in the model group. The overall mortality rates in the vehicle group, ConA group, and Nec-1 group were 30%, 27%, and 7%, respectively. The difference between the Nec-1 group and ConA group was statistically significant (*P* < 0.05), but there was no significant difference between the vehicle group and the ConA group (*P* > 0.05). This suggests that Nec-1 reduced the death rate in mice with ConA-induced acute liver injury (see Table 3).

### 3.2. Nec-1 Pretreatment Protects Mice from ConA-Induced Liver Injury

**Figure 1:**
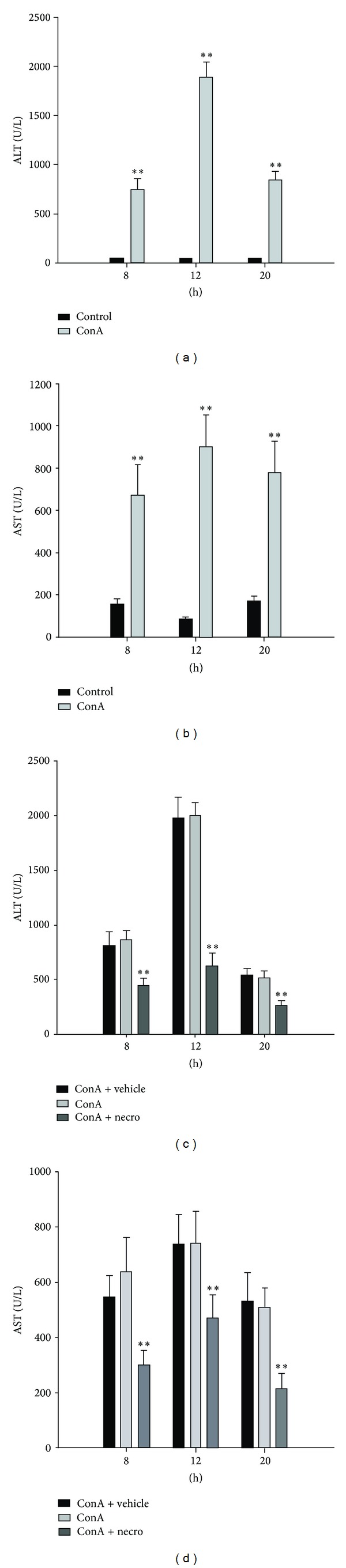
Induction of liver injury by ConA in model mice and Nec-1-treated mice. ((a)-(b)) Plasma ALT and AST levels in mice at 8, 12, and 20 h after ConA injection. Data are expressed as means ± SD (*n* = 10 mice, **P < 0.01). ((c)-(d)) Serum ALT and AST levels were measured at 8, 12, and 20 h after ConA injection in the vehicle-treated, control, and Nec-1-treated mice. Each value is a mean ± SD (*n* = 10 mice, **P < 0.01).

 To determine the protective effects of Nec-1 against ConA-induced liver injury, Nec-1 was administered intraperitoneally to mice 1 h before injection with ConA. First, we established a mouse model of acute liver injury using the tail vein injection of ConA (20 mg/kg). Serum ALT and AST levels and liver tissue biopsies were examined as indicators of liver injury. At 8, 12, and 20 h after injection with ConA, serum ALT and AST levels had changed dynamically (ALT: 810.8 ± 340.9 U/L at 8 h to 840.8 ± 91.7 U/L at 20 h, with a peak of 1888.4 ± 155.9 U/L at 12 h; AST: 667.6 ± 149.9 U/L at 8 h to 778.2 ± 150.5 U/L at 20 h, with a peak of 902.4 ± 150.8 U/L at 12 h) (see Table 4 and Figures 1(a) and 1(b)). 

Serum ALT and AST levels were significantly elevated in the mice after the administration of ConA, whereas Nec-1 pretreatment significantly attenuated the ConA-induced elevation of serum ALT and AST (see Table 5 and Figures 1(c) and 1(d)).

**Figure 2:**
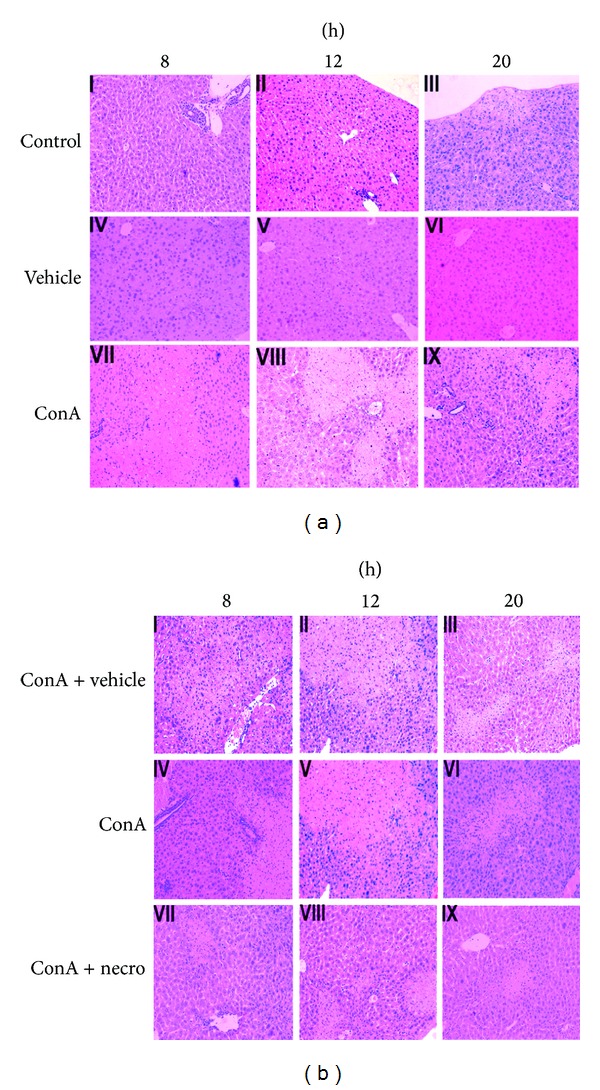
(a) Photomicrographs of representative livers collected 8, 12, and 20 h after ConA injection, stained with H&E (magnification ×200). The control animals received NSS alone ((a) I–III). The vehicle-injected group received DMSO only, without ConA ((a) IV–VI). The mice in the ConA group were injected in the tail vein with ConA for at 8, 12, or 20 h. H&E, ×200 magnification ((a) VII–IX). These experiments were repeated three times, and the same results are shown. (b) Photomicrographs of representative livers collected at 8, 12, and 20 h after ConA injection, stained with H&E (magnification ×200). The vehicle group was intraperitoneally injected with DMSO 1 h before ConA challenge ((b) I–III). ConA-injected control animals received ConA only ((b) IV–VI). The Nec-1-pretreated group was intraperitoneally injected with Nec-1 1 h before ConA challenge ((b) VII–IX). These experiments were repeated three times with the same results.

 To examine the histological changes in the liver after ConA injection in the presence or absence of Nec-1, we stained the liver tissues with H&E. At 8, 12, and 20 h after ConA injection, the hepatic lobules showed inflammatory cells infiltrating the confining ducts, large numbers of infiltrating mononuclear lymphocytes, and a proportion of necrotic liver cells in ConA-induced liver injury model groups (Figure 2(a) VII–IX). Flaky necrosis was visible 20 h after the ConA injection with a pathological examination of the liver tissues (Figure 2(a) IX). These results show that the ConA-treated model was successful. Histopathological studies of the mouse livers were used to determine the effects of Nec-1 on ConA-induced liver injury. As shown in Figure 2(b), a light microscopy examination revealed extensive inflammatory infiltration and large areas of necrosis in the livers of the ConA-treated mice. In contrast, mice pretreated with Nec-1 showed minor liver damage (Figure 2(b) VII–IX). Nec-1 pretreatment markedly increased the survival of the mice. Overall, Nec-1 pretreatment protected the mice from ConA-induced liver injury.

### 3.3. Nec-1 Pretreatment Inhibits the Release of Cytokines during ConA-Induced Hepatitis

**Figure 3:**
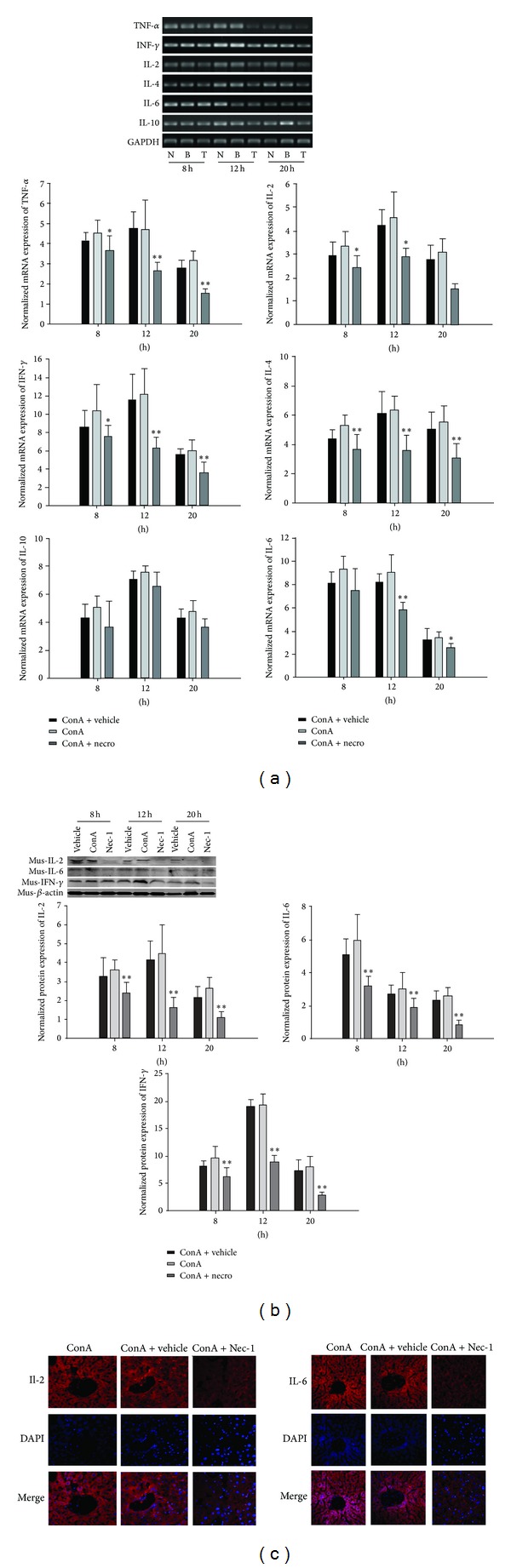
(a) ConA induced the release of cytokines in untreated (blank), negative-control-treated, and Nec-1-treated mice. IL2, IL4, IL6, IL10, TNF-*α*, IFN-*γ*, and GAPDH mRNA expression in the livers of untreated (blank), negative-control-treated, and Nec-1-treated mice. IL2, IL6, IL10, TNF-*α*, and IFN-*γ* mRNA expression in the livers of untreated (blank) and negative-control-treated groups were significantly increased after ConA injection compared with those in the Nec-1-treated mice. (b) Effects of Nec-1 on ConA-induced hepatic IFN-*γ*, IL2, and IL6 protein expression. (c) Localization of IL2 and IL6 in the liver tissues detected with immunofluorescence (original magnification ×200). These experiments were repeated three times, and representative results are shown.

 We conducted a detailed analysis of the mRNA expression of intrahepatic cytokines (IL2, IL4, IL6, IL10, TNF-*α*, and IFN-*γ*) in untreated (blank), vehicle-treated, ConA-treated, and Nec-1-treated mice using PCR. Our results show that, in the ConA-treated model mouse liver tissues, TNF-*α*, IFN-*γ*, IL2, IL4, and IL6 mRNA expression was significantly higher than in the blank control group. After the Nec-1 intervention, the expression of these cytokines (TNF-*α*, IFN-*γ*, IL2, IL4, and IL6) was significantly reduced compared with that in the model group (*P* < 0.05), but the expression of IL10 did not differ significantly (*P* > 0.05) from that in the model group (Figure 3(a)). Thus, ConA-induced hepatic injury is associated with changes in the levels of inflammatory cytokines. The levels of proinflammatory cytokines such as IFN-*γ*, IL2, and IL6 in the liver tissues were assayed by western blotting after the administration of ConA to untreated (blank), DMSO-pretreated, and Nec-1-pretreated mice. Western blotting showed that IFN-*γ*, IL2, and IL-6 protein expression in the model group showed upward trends, whereas their expression was lower in the Nec-1 intervention group, and the differences were statistically significant (*P* < 0.05; Figure 3(b)). As shown in Figure 3(c), immunofluorescence showed that the expression of IL2 and IL6 was significantly lower in the Nec-1 intervention group than in the untreated and DMSO-pretreated model group.

### 3.4. Nec-1 Inhibits RIP1 in ConA-Induced Hepatitis

**Figure 4:**
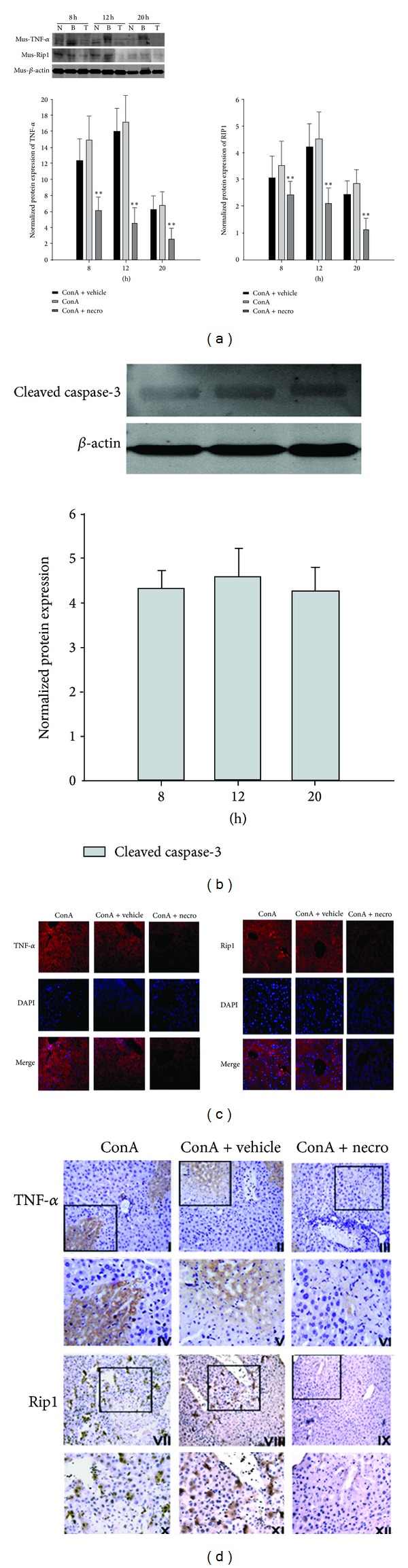
(a) Effects of Nec-1 on ConA-induced hepatic TNF-*α* and RIP1 protein expression. (b) Caspase 3 activity in cytosolic extracts 12 h after ConA-induced liver injury and pretreatment with vehicle or Nec-1 versus ConA model mice. (c) Localization of IL2 and IL6 in liver tissues detected by immunofluorescence (original magnification: ×200). (d) Immunohistochemical staining for RIP1 and TNF-*α* after ConA-induced hepatitis in the negative control group (I, IV, VII, and X), the vehicle group (II, V, VIII, and XI), and the Nec-1-treated group (III, VI, IX, and XII). These experiments were repeated three times, and representative results are shown. The small image is ×200 magnification, and the enlarged pictures show the framed parts in the small images (×400).

 A previous study demonstrated that RIP1 plays a critical role in necroptosis. Its serine/threonine kinase activity is essential for the necrotic death pathway but is not required for either NF-*κ*B activation or apoptosis, which rely on the intermediate and death domains of the protein [30]. The small-molecule inhibitor of necroptosis, Nec-1, is a potent inhibitor of RIP1 kinase activity [19]. Therefore, we investigated whether Nec-1 affects hepatic RIP1 expression, using western blotting and immunocytochemical staining in mice with ConA-induced liver injury. The hepatic expression of RIP1 protein was examined at 8, 12, and 20 h after the ConA challenge. As shown in Figure 4(a), RIP1 protein expression was significantly upregulated at 12 h in the ConA-treated group and was clearly downregulated in the Nec-1-pretreated group. Furthermore, as shown in Figure 4(d), the RIP1 levels in the Nec-1 group were significantly reduced in the cytoplasm of the hepatocytes and in the area of necrosis 12 h after ConA injection compared with those in the negative control and vehicle groups. These data suggest that Nec-1 pretreatment inhibits RIP1 release and expression, thus protecting the mice from ConA-induced hepatic injury. It is well known that RIP1 is more strongly expressed after ConA-induced liver injury. Therefore, we examined the changes in RIP1 in the liver tissues collected from the three experimental groups after 12 h, using immunofluorescence. The results are shown in Figure 4(c). RIP1 was clearly localized in the cytoplasm and was expressed at low levels in the Nec-1 group.

ConA-induced hepatic injury is associated with changes in inflammatory cytokines. Therefore, the levels of proinflammatory cytokines such as TNF-*α* in the liver tissues were assayed by western blotting after the administration of ConA to the model and Nec-1-pretreated mice. As shown in Figure 4(a), the increase in intrahepatic levels of TNF-*α* in response to ConA was prevented by pretreatment with Nec-1. Therefore, the prevention of ConA-induced liver injury by Nec-1 is associated with the inhibition of the release of proinflammatory cytokines, such as TNF-*α*.

Cleavage by caspase 3 was measured to establish the extent of hepatocyte apoptosis. As shown in Figure 4(b), hepatocyte apoptosis was observed in the livers of mice treated with ConA. Nec-1 pretreatment did not dramatically attenuate ConA-induced apoptosis in the liver. These data suggest that Nec-1 pretreatment does not affect hepatocyte apoptosis in ConA-induced hepatitis but confirm that necroptosis and apoptosis are two different types of programmed cell death.

### 3.5. Influence of Nec-1 on Autophagy in ConA-Induced Hepatitis

**Figure 5:**
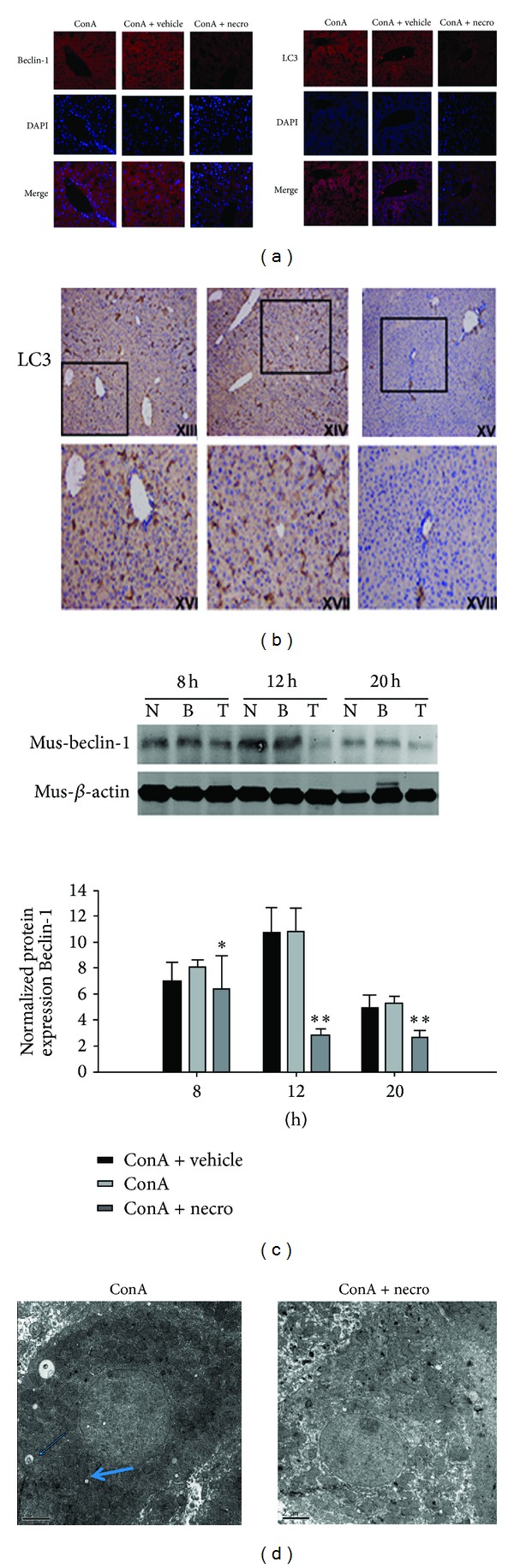
(a) Localization of beclin-1 and LC3 in liver tissues detected with immunofluorescence (original magnification ×200). (b) Immunohistochemical staining of LC3 after ConA-induced hepatitis in the control group, the vehicle-treated group, and the Nec-1-treated group. These experiments were repeated three times, and representative results are shown. The small image is ×200 magnification and the enlarged images show the framed parts of the small images (×400). (c) Effects of Nec-1 on ConA-induced hepatic beclin-1 protein expression. (d) Autophagosome formation induced with ConA is detected in hepatocytes with transmission electron microscopy and was significantly reduced by the Nec-1 intervention. Arrows indicate autophagosomes.

Liver tissues with ConA-induced injury exhibited an increased autophagic flux and enhanced conversion of LC3, a marker of the activation of autophagy in liver tissues. ConA induced autophagosome formation in the hepatocytes, which was detected with TEM, and this was significantly reduced by the Nec-1 intervention, as was the expression of the autophagy-related proteins beclin-1 and LC3, detected with immunofluorescence and western blotting (Figures 5(a)–5(c)). 

Electron microscopic assessment of ConA-induced acute liver injury at 12 h showed the ultrastructural morphological changes of liver tissue necrosis. The ConA model group displayed large areas of necrotic disintegration, with uniform nuclear chromatin in the hepatocytes and a small amount of condensation, although the integrity of the nuclear membrane was less clear; there were many mitochondria within the cytoplasm, but the organellar structure was not clear, although phagosomes were visible. The Nec-1 signal in the hepatic nuclear chromatin was homogeneous, with a small amount of agglutination. The membrane integrity was unclear, there were many mitochondria within the cytoplasm, and the structures still had integrity (Figure 5(d)). 

## 4. Discussion

 In this study, we found that ConA-induced liver injury was ameliorated in Nec-1-treated mice and that this amelioration was accompanied by a reduction in the expression of proinflammatory cytokines and RIP1. Serum ALT and AST levels were significantly reduced in the Nec-1-treated mice after injection with ConA compared with those in the model mice. Liver diseases with various etiologies, including viral and autoimmune hepatitis, represent a significant health problem, and, in most cases, there are no effective medical therapies.

The death receptor family plays a critical role in regulating cell numbers and eliminating harmful or virally infected cells. Agonistic stimulation of the death receptors is known to lead to two alternative cell fates: either the activation of NF-*κ*B to promote cell survival or the induction of apoptosis, which leads to cell death. Now, a third pathway, designated “necroptosis” or “programmed necrosis,” has been identified. Interestingly, a death-domain-containing kinase, RIP1, is involved in the mediation of all three pathways, and its kinase activity is specifically involved in regulating necroptosis. The availability of Nec-1, a specific inhibitor of RIP1 kinase, has made it possible to distinguish the distinct functional domains of RIP1 [31].

Necrostatin,1,5-(1H-indol-3-ylmethyl)-(2-thio-3-methyl)-one, a small-molecule inhibitor of programmed cell necrosis, has shown promise as a neuroprotectant in adult rodent models of myocardial ischemia and traumatic or ischemic brain injury [16, 28, 32]. Nec-1 protects cells by inhibiting RIP1 kinase activation, with possible downstream anti-inflammatory effects, and by other mechanisms [19, 28]. It is a polyphenol with potential beneficial health effects in humans. A recent study showed that treatment with Nec-1 reduced necrotic cell death and increased apoptotic cell death. Nec-1 also reduced hypoxia-ischemia-induced oxidative damage to proteins and attenuated markers of inflammation coincidental with reduced activation of nuclear factor-*κ*B and caspase 1 [33]. In vitro, necrostatin has little effect on classic apoptotic cell death but, instead, strongly inhibits programmed cell necrosis [16].

 ConA, a lectin with mannose specificity that can induce acute hepatic inflammation, was tested for its therapeutic effects on hepatoma [34]. Lectins are carbohydrate-binding proteins that occur throughout the biosphere. They bind carbohydrates reversibly and can agglutinate cells or precipitate polysaccharides or glycol conjugates. They have strong mitogenic activity toward lymphocytes [35–37]. ConA-induced hepatitis is a well-known murine model of fulminant hepatitis attributed to excessive immune responses, such as autoimmune hepatitis. Previous studies have shown that the pathogenesis of ConA-induced hepatitis is mainly mediated by the release of inflammatory cytokines. Of those cytokines, TNF-*α* is secreted by hepatic Kupffer cells and acts as a major mediator of inflammation-induced hepatocyte apoptosis when it binds to its receptors [33]. The identification of the RIP1 kinase inhibitor Nec-1 has allowed researchers to identify the involvement of necrotic cell death in an increasing number of pathological conditions. Indeed, RIP1 kinase activity is not essential under most apoptotic conditions, but it is crucial for the activation of the regulated form of necrosis recently designated “necroptosis” [31].

 PCR analysis revealed that the expression of IL2, IL4, IL6, IL10, TNF-*α*, IFN-*γ*, and GAPDH mRNAs in the livers of untreated (blank) and DMSO-treated mice was significantly upregulated 12 h after their injection with ConA (Figure 3). Nec-1 is a small molecule chosen from an extensive chemical library for its ability to inhibit the cell death caused by TNF-*α* stimulation in the context of caspase inhibition [16]. It is an allosteric inhibitor of RIP1 and is a key signaling intermediate in the cell death described as “programmed necrosis” [38]. In vitro, the inhibition of RIP1 kinase blocks the cell death caused by death-receptor signaling in the presence of caspase inhibition [19]. In experimental models of acute liver injury, liver cell death mainly occurs through necrosis and apoptosis, and different models of hepatic inflammation show different apoptosis/necrosis ratios. The severity of the experimental acute inflammation in the liver correlates positively and directly with the degree of liver cell necrosis and correlates negatively with the level of apoptosis, the exact mechanism of which is not yet clear.

 In the present study, there was no significant change in the expression of RIP1 in the first 8 h in the mouse model of acute liver injury induced with ConA, but its expression was significantly upregulated in the first 12 h and 20 h after ConA treatment. Programmed necrosis in acute liver injury and its underlying molecular mechanism have not been clarified. However, He et al. [39] and Zhang et al. [40] reported that, in RIP3^−/−^ mice produced using gene knockout technology, ConA-induced acute liver damage with liver cell necrosis was significantly reduced. RIP1 and RIP3 are two key kinases involved in the regulation of programmed necrosis. Our research and other studies indicate that programmed necrosis may be involved in ConA-induced acute liver injury.

 Nec-1 is a specific chemical inhibitor of programmed necrosis, and has been shown to inhibit the phosphorylation of RIP1, thereby inhibiting RIP1 function and the necrotic process. Nec-1 also protects against hypoxia-ischemia/reperfusion brain damage and inhibits retinal-detachment-caused photoreceptor cell necrosis and retinal ischemia/reperfusion injury [33]. If acute liver injury is a necrosis-related disease, does Nec-1 have a similar protective effect in the liver? To address this question, we investigated the effects of Nec-1 administered by intraperitoneal injection on ConA-induced acute liver injury in vitro.

 We found that Nec-1 significantly reduced the levels of serum transaminases, inhibited liver cell necrosis, reduced the severity of acute liver injury, and confirmed the presence of programmed necrosis. We also found that hepatic TNF-*α* expression was significantly reduced at the same time. Liver cell necrosis and inflammatory responses play an important role in the development of acute liver injury. The necrosis of liver cells is an important source of the inflammatory response and the increased production and release of inflammatory cytokines; Nec-1 does not inhibit TNF-*α*-induced NF-*κ*B activation. Therefore, we speculate that the inhibition of the production of inflammatory mediators by Nec-1 is caused by its reduction of necroptosis during ConA-induced hepatitis. Nec-1 prevents the programmed necrosis of hepatocytes by inhibiting the expression of TNF-*α* and RIP1 in vivo, thereby inhibiting the process of hepatocyte necrosis. This confirms that the Nec-1 intervention can reduce ConA-induced acute liver injury. 

Recently, many studies have shown that autophagy is upregulated during hepatic IR injury [41]. Autophagy is an essential cellular process that mediates continuous recycling of intracellular components [42]. Recent evidence supports the view that enhancing autophagy may be a novel approach to improve hepatocyte viability and function after I/R injury, and such a hepatoprotective role of autophagy may be associated with its antiapoptotic and anti-inflammatory activity [43]. In the present study, we found that autophagy was inhibited by Nec-1 preconditioning in hepatocytes in vitro. These results suggest that autophagy is also a key mediator in the hepatoprotective effect of Nec-1 intervention. 

 In summary, our study is the first to confirm that Nec-1 has a significant protective effect against acute liver injury in mice, suggesting that the programmed necrosis of liver cells may have important clinical implications for the inhibition of acute liver injury, providing new ideas and targets for the treatment of this disease. Nec-1, the role of its targets, and the molecular mechanism of programmed necrosis require further study.

## Figures and Tables

**Table 1:** Nucleotide sequences of primers used for PCR.

Gene	Primer	Sequence (5′-3′)	Melting temperature (°C)	Product size (bp)
TNF-*α*	Forward	CAGGCGGTGCCTATGTCTC	62.2	89
	Reverse	CGATCACCCCGAAGTTCAGTAG	62.1	
IFN-*γ*	Forward	GCCACGGCACAGTCATTGA	62.9	201
	Reverse	TGCTGATGGCCTGATTGTCTT	61.8	
IL-2	Forward	TGAGCAGGATGGAGAATTACAGG	61.4	120
	Reverse	GTCCAAGTTCATCTTCTAGGCAC	60.6	
IL-4	Forward	GGTCTCAACCCCCAGCTAGT	62.8	102
	Reverse	GCCGATGATCTCTCTCAAGTGAT	61.5	
IL-6	Forward	CTGCAAGAGACTTCCATCCAG	60.1	131
	Reverse	AGTGGTATAGACAGGTCTGTTGG	60.8	
IL-10	Forward	CTTACTGACTGGCATGAGGATCA	61.4	101
	Reverse	GCAGCTCTAGGAGCATGTGG	62.4	
GAPDH	Forward	AGGTCGGTGTGAACGGATTTG	62.6	95
	Reverse	GGGGTCGTTGATGGCAACA	62.6	

**Table 2:** Mortality changes in ConA-induced hepatic injury.

Group	Case (*n*)	Number of death (*n*)	Number of survival (*n*)	Mortality (%)
Blank control	24	0	24	0
ConA model	30	6	24	20.0*
8 h	10	1	9	10.0
12 h	10	3	7	30.0
20 h	10	2	8	20.0

*Note*. *Mouse mortality compared with the model and control groups, *P* < 0.05.

**Table 3:** Influence of the Nec-1 intervention on mortality caused by ConA-induced liver injury.

Groups	Case (*n*)	Number of death (*n*)	Number of survival (*n*)	Mortality (%)
Vehicle	30	9	21	30
ConA model	30	8	22	27.0
Nec-1 intervention	30	2	28	7.0*

*Note*. *Mortality compared between the Nec-1, ConA, and vehicle groups, *P* < 0.05.

**Table 4:** Changes in liver function in ConA-induced hepatitis (means ± SD).

Time (H)	Number of animals (*n*)	ALT (U/L)	AST (U/L)
Blank control	10	36.2 ± 4.2	154.9 ± 27.2
8 h model group	10	810.8 ± 340.9*	667.6 ± 149.9^#^
12 h model group	10	1888.4 ± 155.9*	902.4 ± 150.8^#^
20 h model group	10	840.8 ± 91.7*	778.2 ± 150.5^#^

*Notes*. ALT compared with the model and control groups, *P < 0.05; AST compared with the model and control groups, ^#^ *P* < 0.05.

**Table 5:** Changes in liver function in ConA-induced hepatitis after the Nec-1 intervention (means ± SD).

Groups	Number of animals (*n*)	ALT (U/L)			AST (U/L)		
		8 h	12 h	20 h	8 h	12 h	20 h
Vehicle	30	803 ± 133	1972 ± 199	535 ± 68	502 ± 158	733 ± 110	528 ± 107
ConA model	30	857 ± 92	1996 ± 126	507 ± 72	506 ± 232	730 ± 117	507 ± 72
Nec-1 intervention	30	441 ± 68*	616 ± 128*	259 ± 47*	219 ± 72^#^	461 ± 90^#^	212 ± 58^#^

*Notes*. ALT compared with the Nec-1-, vehicle-, and ConA-treated groups, *P < 0.05; AST compared with the Nec-1-, vehicle-, and ConA-treated groups, ^#^ *P* < 0.05.
